# Sodiophilic Na_3_Sb Framework‐Regulated Sodium Deposition and Interphase Evolution for Dendrite‐Free and Long‐Life Sodium Metal Batteries

**DOI:** 10.1002/advs.77935

**Published:** 2026-09-27

**Authors:** So Hyun Lee, Dawon Baek, Inkyung Kim, Sang Jun Lee, Ho Jin Ma, Tae Hoon Hwang, San Moon, Joo‐Hyung Kim

**Affiliations:** ^1^ Department of Materials Science and Engineering Gyeongsang National University (GNU) Jinju Republic of Korea; ^2^ Department of Materials Engineering and Convergence Technology Gyeongsang National University (GNU) Jinju Republic of Korea; ^3^ Department of Chemical and Biomolecular Engineering Korea Advanced Institute of Science and Technology (KAIST) Daejeon Republic of Korea; ^4^ Department of Advanced Materials Engineering Kyonggi University Suwon Republic of Korea; ^5^ Battery Development Center – Battery Electrode System Research Lab Hyundai Motor Company Co., Ltd Uiwang Republic of Korea; ^6^ Department of Advanced Battery Research Center Korea Research Institute of Chemical Technology (KRICT) Daejeon Republic of Korea

**Keywords:** 3D framework, dendrite‐free, NaF‐rich, Na‐Sb alloy, sodiophilicity, sodium metal batteries

## Abstract

Sodium (Na) metal anodes are attracting great attention as next‐generation energy storage systems due to their high theoretical capacity, abundant resources, and low cost. However, an unstable solid‐electrolyte interphase (SEI), uncontrolled dendrite growth, and large volume changes are major factors limiting the practical application of Na metal anodes. Herein, we develop a Na‐Sb alloy anode featuring a three‐dimensional sodiophilic Na_3_Sb framework embedded within a metallic Na matrix. The interconnected Na_3_Sb framework homogenizes the Na^+^ flux and provides abundant Na nucleation sites, enabling uniform Na deposition while effectively suppressing dendrite growth. Moreover, the engineered interface promotes the formation of a dense, NaF‐rich inorganic SEI with high mechanical strength and chemical stability, which effectively accommodates volume changes and stabilizes the Na metal/electrolyte interface during repeated cycling. Consequently, the NSF‐3||NSF‐3 symmetric cell delivers remarkably stable cycling for over 1000 h at 0.5 mA cm^−2^ with a capacity of 0.5 mAh cm^−2^, exhibiting significantly improved interfacial stability and Na plating behavior. This work demonstrates that constructing a sodiophilic Na_3_Sb framework is an effective strategy for regulating Na deposition and stabilizing the Na metal interface, providing a practical pathway toward high‐energy‐density and long‐life sodium metal batteries.

## Graphical abstract

An interconnected sodiophilic Na_3_Sb framework embedded within a metallic Na matrix enables uniform Na deposition by homogenizing Na^+^ flux and providing abundant nucleation sites. Simultaneously, the regulated interface promotes the formation of a stable, NaF‐rich inorganic interphase, collectively suppressing dendrite growth and enabling highly reversible, long‐term Na plating/stripping for durable sodium metal batteries.

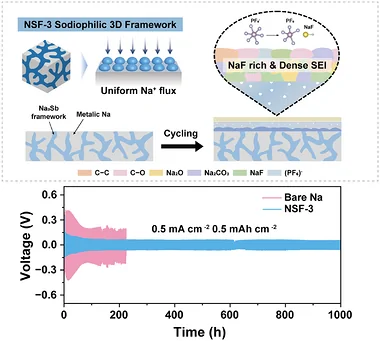

## 1 Introduction

Sodium (Na)‐based batteries, which are abundant in resources and highly cost‐effective, are gaining attention as a promising energy storage system for contributing to a sustainable future. Due to its ultra‐high theoretical specific capacity (∼1166 mAh g^−1^) and low redox potential (−2.71 V vs. SHE), Na metal is considered an ideal anode material for realizing high‐energy‐density sodium metal batteries (SMBs) [1, 2, 3, 4, 5, 6, 7]. Despite these advantages, Na metal anodes still face challenges in achieving reversible and stable Na plating/stripping performance during long‐term cycling. This is mainly attributed to the high reactivity of Na metal and the mechanically fragile solid‐electrolyte interface (SEI), which cannot effectively buffer the volume changes occurring during the Na plating/stripping process, resulting in uneven Na plating and dendrite growth. The growth of Na dendrites leads to the accumulation of inactive Na (dead Na) and continuous electrolyte consumption, thereby degrading coulombic efficiency (CE) and cycling stability. Furthermore, Na dendrites can penetrate the separator and cause internal short circuits, posing serious safety concerns and representing a critical technical challenge for the practical application of SMBs [8, 9, 10, 11, 12, 13, 14, 15]. To address these issues, various strategies have been investigated in recent years. These include electrolyte optimization [16, 17], the formation of an artificial SEI [18, 19, 20], the application of solid electrolytes [21, 22], and methods involving the introduction of metal‐based three‐dimensional (3D) Na hosts [23, 24, 25].

In particular, the introduction of alloy‐based 3D hosts can regulate the Na^+^ flux due to their sodiophilic properties, thereby inducing uniform Na plating, inhibiting dendrite formation, mitigating volume expansion, and reducing side reactions with the electrolyte [26, 27]. In addition, alloy‐based hosts exhibit great potential for achieving high energy density owing to their high reversible capacity through alloying reactions [28]. Nevertheless, the practical application of alloy‐type Na hosts remains challenging because each alloy system possesses intrinsic limitations associated with cost, specific capacity, or structural instability. For example, Ye et al. [29] proposed a 3D‐Na_3_Bi alloy host structure to induce uniform nucleation and growth of Na metal. The structure, in which electronic and ionic conductivity alternate periodically, enhances mass transfer rates, relieving volume expansion and alleviating dendrite. Bi‐based alloy hosts exhibit excellent sodiophilicity and rapid Na diffusion kinetics, but their relatively high material cost and low theoretical capacity (∼385 mAh g^−1^) limit their practical energy density and large‐scale applicability. Moreover, the large atomic weight of Bi inevitably reduces gravimetric energy density despite its favorable interfacial characteristics. Moreover, Li et al. [30] proposed a Na_15_Sn_4_/Na composite alloy foil utilizing the spontaneous alloying reaction between metallic Na and Sn. The formed Na_15_Sn_4_ alloy skeleton regulated Na plating/stripping performance and induced uniform Na plating, thereby inhibited the Na dendrite growth. As a result, the symmetric cell maintained a low overpotential (∼15 mV) even under conditions of 60°C, 1 mA cm^−2^, and 1 mAh cm^−2^, exhibiting stable cycling performance for over 100 cycles, confirming improved electrochemical performance compared to cells using a pristine Na anode. Despite these advantages, Sn‐based alloy hosts remain limited by severe volume expansion and structural instability during repeated sodiation/desodiation. In particular, the formation of highly sodiated phases such as Na_15_Sn_4_ generates substantial internal stress, leading to particle pulverization, structural collapse, unstable SEI formation, and continuous side reactions caused by repeated exposure of fresh surfaces to the electrolyte.

To overcome the limitations of previously reported Na‐transition metal alloy hosts, Sb has emerged as a promising alloy component owing to its high Na affinity, high theoretical capacity, relatively low operating potential, and lower volume expansion compared with Sn‐based systems. In particular, the favorable sodiophilic nature of Sb can effectively regulate Na nucleation and deposition behavior, while its relatively moderate volume change contributes to improved structural stability during repeated cycling. These characteristics suggest that Sb‐based alloy hosts can simultaneously address the interfacial instability and mechanical degradation commonly encountered in conventional alloy‐type Na hosts, making them attractive candidates for stable Na metal anodes.

In this study, a Na‐Sb alloy host was readily fabricated through a simple metallurgical process, resulting in a structure in which a Na_3_Sb‐based framework is uniformly distributed throughout the Na metal matrix. The formed Na_3_Sb framework provides physically stable Na deposition sites and effectively alleviates local current density, thereby promoting homogeneous Na deposition behavior. Furthermore, the Na_3_Sb framework chemically induces the formation of a stable inorganic‐rich SEI layer during repeated Na plating/stripping processes, contributing to enhanced interfacial stability and suppressed side reactions with the electrolyte. As a result, the fabricated Na‐Sb alloy host enables the formation of a dense and uniform Na deposition layer while effectively mitigating volume fluctuation and interfacial instability during cycling. Collectively, these characteristics suggest that the proposed Na‐Sb alloy framework can serve as a highly promising strategy for realizing stable and reliable Na metal anodes for next‐generation SMBs.

## 2 Results and Discussion

**FIGURE 1:**
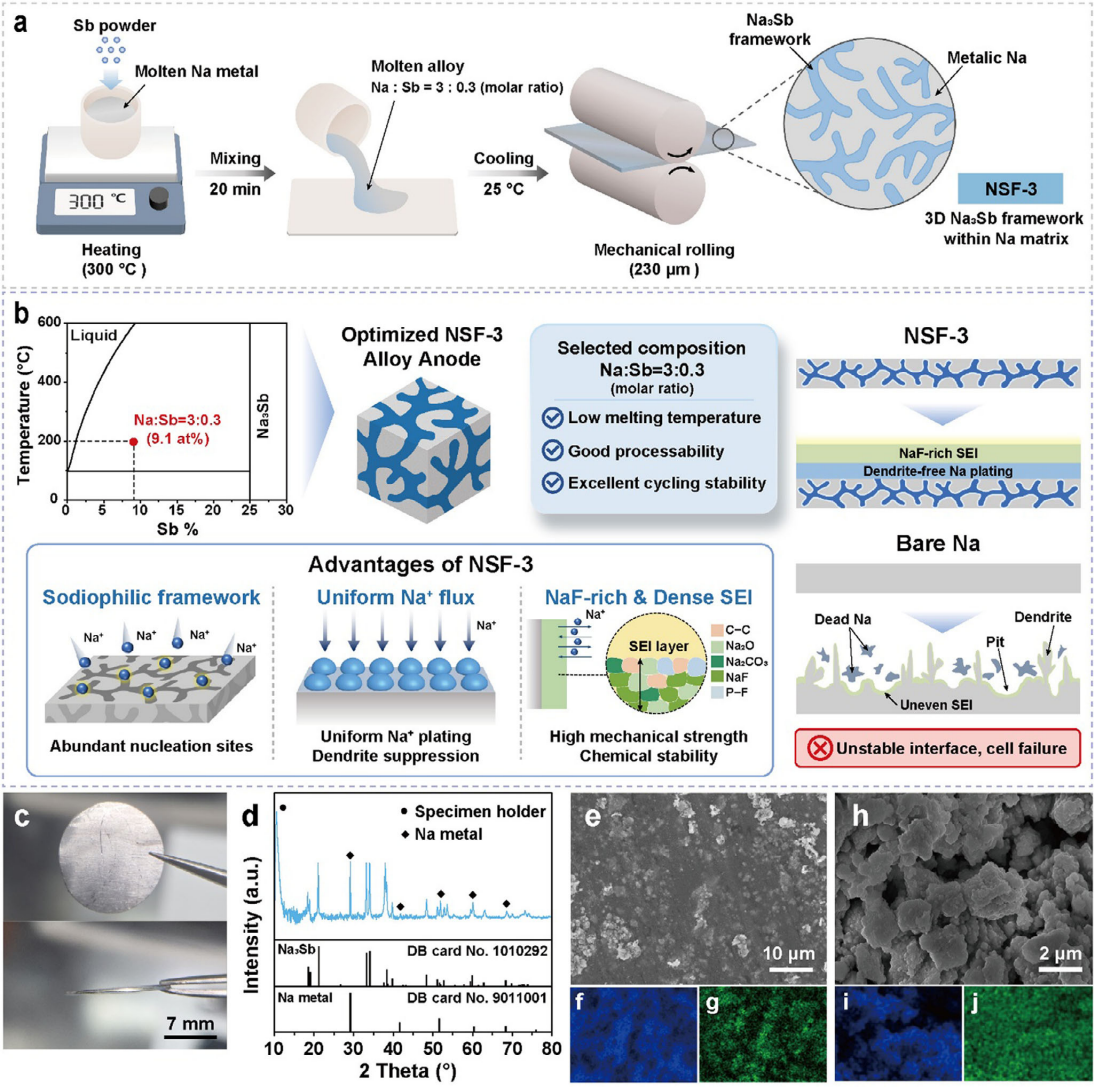
Schematic illustration and structural characterization of the NSF‐3 electrode. (a) Schematic illustration of the synthesis process for NSF‐3(Na:Sb = 3:0.3, molar ratio). (b) Structural advantages and interfacial stabilization mechanism of the optimized NSF‐3. (c) Photograph of a 14 mm diameter NSF‐3. (d) XRD pattern of NSF‐3. (e) Top‐view SEM image of NSF‐3 and the corresponding EDS elemental maps of (f) Na and (g) Sb. (h) SEM image of the electrode after Na stripping from NSF‐3 and the corresponding EDS elemental maps of (i) Na and (j) Sb.

Figure 1a illustrates the fabrication strategy for the Na–Sb composite anode (NSF‐3). Metallic Na was first alloyed with Sb through a melt‐alloying process, followed by a mechanical rolling process to produce thin electrodes with a thickness of approximately 250 µm. The combination of melt alloying and rolling provides a simple and scalable route for the large‐area fabrication of Na metal composite anodes. Based on the Na–Sb phase diagram, the selected Na molar ratio of 3:0.3 results in the coexistence of metallic Na and Na_3_Sb phases. Assuming complete alloying of Sb with Na, the composition corresponds to approximately 0.3 mol Na_3_Sb and 2.1 mol residual metallic Na, yielding a Na_3_Sb molar ratio of about 1:7. Consequently, NSF‐3 consists predominantly of a metallic Na matrix containing a relatively small fraction of Na_3_Sb that is homogeneously distributed throughout the electrode. Figure 1b outlines the basis for selecting NSF‐3 as the optimal composition and illustrates the structural advantages and stabilization mechanism of the alloy electrode. Na:Sb = 3:0.x (x = 1, 2, 3, 4) was fabricated using the same method as in Figure 1a. As the Sb content increased, the metallic luster of the electrode surface gradually decreased, and the color became relatively darker (Figure S1). The Na–Sb alloy system offers a relatively low melting temperature and good processability, making it suitable for the fabrication of uniform alloy electrodes. Considering the intrinsic physical properties of metallic Na and Na_3_Sb, NSF‐3 was considered to provide an appropriate balance between the sodiophilic Na_3_Sb framework and the processability of the Na matrix. The representative physical properties of Na and Na_3_Sb relevant to the electrode design are summarized in Table S1. The optimized NSF electrode is expected to provide a continuous sodiophilic alloy framework with abundant Na nucleation sites, thereby regulating the Na^+^ flux and promoting uniform Na plating. Such a structural design is anticipated to effectively suppress dendrite growth. Moreover, the optimized interfacial chemistry is expected to facilitate the formation of a dense, NaF‐rich SEI with high mechanical strength and chemical stability, ultimately contributing to enhanced long‐term cycling stability.

The rolled NSF‐3 electrode could be readily punched into 14 mm diameter discs for electrochemical evaluation (Figure 1c). The x‐ray diffraction (XRD) pattern of NSF‐3 (Figure 1d) exhibits diffraction peaks corresponding exclusively to metallic Na and Na_3_Sb phases. Compared with the diffraction pattern of the pristine Sb powder (Figure S2a), no characteristic reflections of elemental Sb were observed, suggesting complete conversion of Sb into the Na–Sb alloy phase during the fabrication process. These results confirm the successful formation of a biphasic NSF‐3 composite without detectable residual Sb. Surface morphology and elemental distribution were further investigated by scanning electron microscopy (SEM) and energy‐dispersive spectroscopy (EDS). While pristine Na metal (Bare Na) exhibited a relatively smooth and featureless surface (Figure S3), NSF‐3 displayed a distinct microstructure arising from the incorporation of Na_3_Sb (Figure 1e–g). Elemental mapping confirmed a uniform distribution of Sb throughout the electrode surface. This observation was further corroborated by backscattered electron (BSE) imaging (Figure S4), where the brighter Na_3_Sb domains were evenly dispersed within the darker Na matrix. The homogeneous distribution of the minor Na_3_Sb phase suggests the formation of abundant sodiophilic sites throughout the electrode, which are expected to regulate Na nucleation and deposition behavior during battery operation.

Figures S5 and S6a show the time–voltage profiles of the Bare Na and NSF‐X (X = 1, 2, 3, 4) electrodes. The voltage polarization values after cycling are summarized in Table S2. NSF‐3 maintained low and stable voltage polarizations of approximately 132, 73, and 72 mV after 30, 150, and 300 cycles, respectively. Furthermore, the nucleation overpotential for the Bare Na, NSF‐1, NSF‐2, NSF‐3, and NSF‐4 electrodes was 921, 617, 653, 518, and 450 mV, respectively (Figure S6b). All NSF‐X electrodes exhibited substantially lower nucleation overpotential than Bare Na, and the overall decrease in nucleation overpotential with increasing Sb content indicates that the increased Na3Sb fraction provides more sodiophilic sites for Na nucleation. In particular, NSF‐4 exhibited the lowest nucleation overpotential, indicating favorable initial Na nucleation behavior. However, the enhanced initial nucleation behavior did not directly translate into superior long‐term cycling stability. Compared with NSF‐4, NSF‐3 maintained lower and more stable polarization during prolonged cycling. At the same time, the higher Na_3_Sb fraction in NSF‐4 increased the stiffness of the electrode and reduced its processability, making it more difficult to obtain a uniform electrode during fabrication. In contrast, NSF‐3 provided sufficient sodiophilic Na_3_Sb sites for favorable Na nucleation while maintaining relatively uniform electrode formation and suitable processability. Therefore, NSF‐3 was selected as the optimized composition based on the combined consideration of Na nucleation behavior, long‐term plating/stripping stability, and electrode processability.

The structural robustness of NSF‐3 was investigated to clarify the role of the Na_3_Sb framework. To this end, metallic Na was selectively removed from the composite electrode through an electrochemical stripping process, followed by SEM, EDS, and XRD analyses (Figure 1h–j and Figure S7a,b). In the SEM images taken after Na stripping, a framework structure corresponding to Na_3_Sb was clearly observed. XRD analysis was performed to confirm the composition of this framework. The results showed no distinct diffraction peaks attributable to residual Na or Sb. Only diffraction peaks corresponding to Na_3_Sb were observed. To more clearly confirm the spatial distribution of the Na_3_Sb framework within the electrode, low‐and high‐magnification cross‐sectional SEM images are presented in Figure S7c,d, respectively. The cross‐sectional SEM images showed that the Na_3_Sb framework was broadly distributed not only on the electrode surface but also throughout the interior of the electrode along the thickness direction. In addition, X‐ray photoelectron spectroscopy (XPS) analysis of the O 1s/Sb 3d spectrum of the NSF‐3 surface showed no detectable Sb 3d5/2 peak corresponding to metallic Sb at approximately 528 eV, further supporting the absence of residual metallic Sb on the NSF‐3 surface (Figure S8). These results clearly demonstrate that the fabricated NSF‐3 electrode consists of a 3D Na_3_Sb framework formed within a Na matrix. After Na extraction, the diffraction peaks corresponding to metallic Na completely disappeared, whereas the characteristic reflections of Na_3_Sb remained unchanged. These results confirm that a continuous Na_3_Sb framework persists even after substantial Na removal, demonstrating its ability to maintain the structural integrity of the electrode. Such a framework is expected to provide mechanically stable sodiophilic sites and preserve continuous electron transport pathways during repeated Na plating/stripping processes. Therefore, NSF‐3 proves to be the most optimized composition in terms of Na plating stability, nucleation behavior, and long‐term cycling stability compared to Bare Na and other NSF‐X electrodes.

**FIGURE 2:**
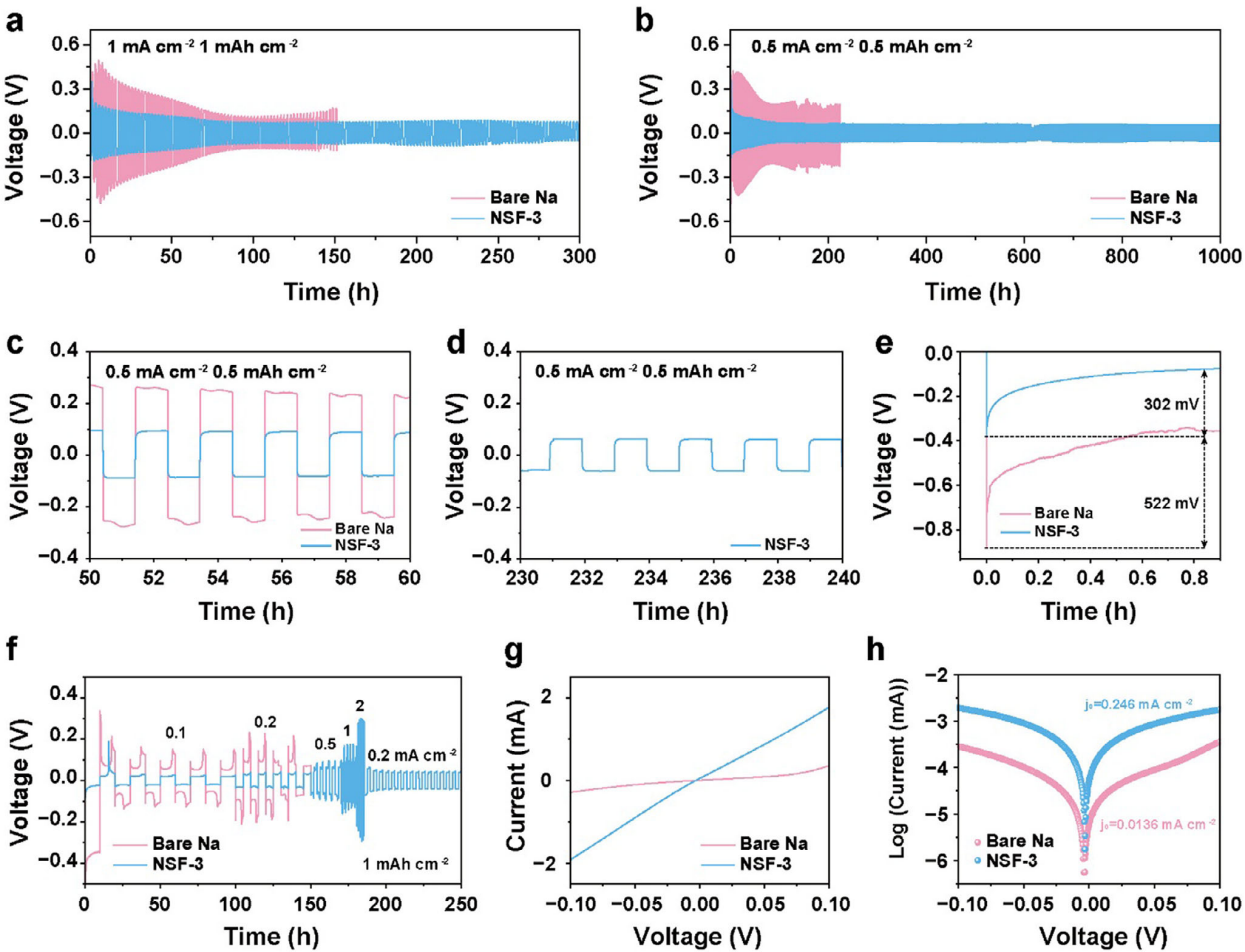
Electrochemical performance of NSF‐3||NSF‐3 and Bare Na||Bare Na symmetric cells. Time‐voltage profiles of NSF‐3 anode and Bare Na anode (a) at a current density of 1 mA cm^−2^ with a capacity of 1 mAh cm^−2^, (b) at a current density of 0.5 mA cm^−2^ with a capacity of 0.5 mAh cm^−2^, and (c, d) their zoomed‐in voltage profile at different cycling states. (e) Nucleation overpotential profile of NSF‐3 and Bare Na. (f) Rate performance of NSF‐3 at a capacity of 0.1, 0.2, 0.5, 1, 2 mA cm^−2^ at a fixed current density of 1 mAh cm^−2^. (g) Comparison of the LSV slopes of Bare Na and NSF‐3 symmetric cells after 20 cycles. (h) Exchange current densities (j_0_) calculated from the Tafel plots of Bare Na and NSF‐3 symmetric cells after 20 cycles.

The electrochemical Na plating/stripping behavior of NSF‐3 was subsequently evaluated in symmetric cells and compared with that of pristine Na metal (Bare Na). All measurements were conducted using Bare Na||Bare Na and NSF‐3||NSF‐3 symmetric configurations. Figure 2a,b present the voltage profiles obtained at current densities/capacities of 1 mA cm^−2^/1 mAh cm^−2^ and 0.5 mA cm^−2^/0.5 mAh cm^−2^, respectively. Under these conditions, the Bare Na cells suffered from short‐circuit failure after 151 and 234 h, respectively (Figure S9a,b), indicating unstable Na deposition and progressive dendrite growth. In contrast, the NSF‐3 cells exhibited remarkably prolonged cycling stability, maintaining stable operation for over 300 and 1000 h with low voltage hysteresis of approximately 71 and 54 mV, respectively (Figure S9c,d). A similar trend was also observed under a capacity of 1.0 mAh cm^−2^ at a current density of 1.0 mA cm^−2^ (Figure S10). Consistently, a significant reduction in polarization was also observed throughout the cycling process. After the initial stabilization period (∼50 h), the NSF‐3 cells exhibited voltage polarizations of approximately 110 and 90 mV, respectively, whereas the corresponding Bare Na cells showed substantially higher values of approximately 210 and 250 mV (Figure 2c and Figure S10a). Even during prolonged cycling (∼240 h), the NSF‐3 cells maintained low polarization values of approximately 85 and 63 mV, indicating highly reversible Na deposition and stripping behavior (Figure 2d). Even under the increased areal capacity condition of 1 mAh cm^−2^ at a current density of 0.5 mA^−2^, NSF‐3 maintained a stable voltage profile with low polarization for more than 1000 h, whereas Bare Na exhibited rapid cell failure (Figure S11), further demonstrating the effectiveness of the embedded Na_3_Sb framework in regulating Na deposition and suppressing dendrite‐induced short circuits over a wide range of operating conditions.

Figure 2e presents the initial Na plating profiles used to evaluate the nucleation behavior of the electrodes. The measurements were performed at a current density of 1 mA cm^−2^ with a plating capacity of 1 mAh cm^−2^. The nucleation overpotential of NSF‐3 was approximately 302 mV, which is substantially lower than that of Bare Na (523 mV). The reduced nucleation overpotential suggests a lower energy barrier for Na nucleation on the NSF‐3 surface, indicating more favorable nucleation kinetics and a greater tendency for homogeneous Na deposition. The rate capability of the symmetric cells was further investigated by increasing the current density from 0.1 to 2 mA cm^−2^ while maintaining a fixed capacity of 1 mAh cm^−2^ (Figure 2f). Throughout the entire current density range, NSF‐3 exhibited stable voltage profiles with relatively low polarization values of approximately 21, 41, 93, 170, and 300 mV at 0.1, 0.2, 0.5, 1, and 2 mA cm^−2^, respectively. In contrast, Bare Na experienced cell failure accompanied by short‐circuit behavior during the rate test. Even at a high current density of 2 mA cm^−2^, NSF‐3 maintained stable cycling behavior without abrupt voltage fluctuations. When the current density was subsequently returned to 0.2 mA cm^−2^, only a slight increase in polarization (∼4 mV) was observed compared with the initial cycle at the same current density, demonstrating excellent reversibility and structural robustness.

To further investigate the evolution of interfacial reaction kinetics during cycling, linear sweep voltammetry (LSV) and Tafel analyses were performed on Bare Na and NSF‐3 symmetric cells at different cycling states. The initial‐state LSV and Tafel results are provided in Figure S12. Additional LSV measurements were performed after 5, 10, and 20 cycles to examine the interfacial charge‐transfer behavior during repeated Na plating/stripping (Figure 2g and Figure S13a,b). The slope obtained from the linear region of the LSV curve is inversely proportional to the interfacial resistance. After 20 cycles, NSF‐3 exhibited a larger LSV slope of 0.03106 than Bare Na (0.01895), indicating substantially reduced interfacial resistance and more facile charge‐transfer processes. NSF‐3 also consistently exhibited higher LSV slopes than Bare Na during cycling, demonstrating that favorable interfacial charge‐transfer kinetics were maintained during repeated Na plating/stripping. The Na plating/stripping kinetics were further evaluated using Tafel analysis after 5, 10, and 20 cycles (Figure 2h and Figure S13c,d). The exchange current density (j_0_), which reflects the intrinsic charge‐transfer kinetics at the electrode/electrolyte interface, was calculated to be 0.333 mA cm^−2^ for NSF‐3 after 20 cycles, markedly higher than that of Bare Na (0.092 mA cm^−2^). Moreover, the exchange current density of Bare Na decreased with cycling, whereas that of NSF‐3 increased. The significantly higher j_0_ value of NSF‐3 indicates more facile charge‐transfer processes and improved Na plating/stripping kinetics in the presence of the Na_3_Sb framework [31]. Cyclic voltammetry (CV) measurements were further performed at scan rates ranging from 0.1 to 5 mV s^−1^ to compare the Na plating/stripping kinetics of Bare Na and NSF‐3 (Figure S14). Across the investigated scan rates, NSF‐3 exhibited a more favorable electrochemical response with reduced polarization compared with Bare Na, indicating faster and more reversible Na plating/stripping kinetics. These results are consistent with the LSV and Tafel analyses, further supporting that the Na_3_Sb framework facilitates interfacial charge transfer and promotes favorable Na plating/stripping kinetics.

The enhanced electrochemical performance of NSF‐3 can therefore be attributed to the uniformly distributed sodiophilic Na_3_Sb framework embedded within the Na matrix. The presence of Na_3_Sb provides abundant energetically favorable nucleation sites for Na deposition, thereby reducing the nucleation barrier and promoting spatially uniform Na‐ion flux. Such regulated Na deposition behavior is expected to mitigate localized current concentration and suppress dendritic Na growth during repeated cycling. As a result, the NSF‐3 electrode exhibits significantly improved cycling stability, lower polarization, and enhanced rate capability compared with pristine Na metal [32, 33, 34].

**FIGURE 3:**
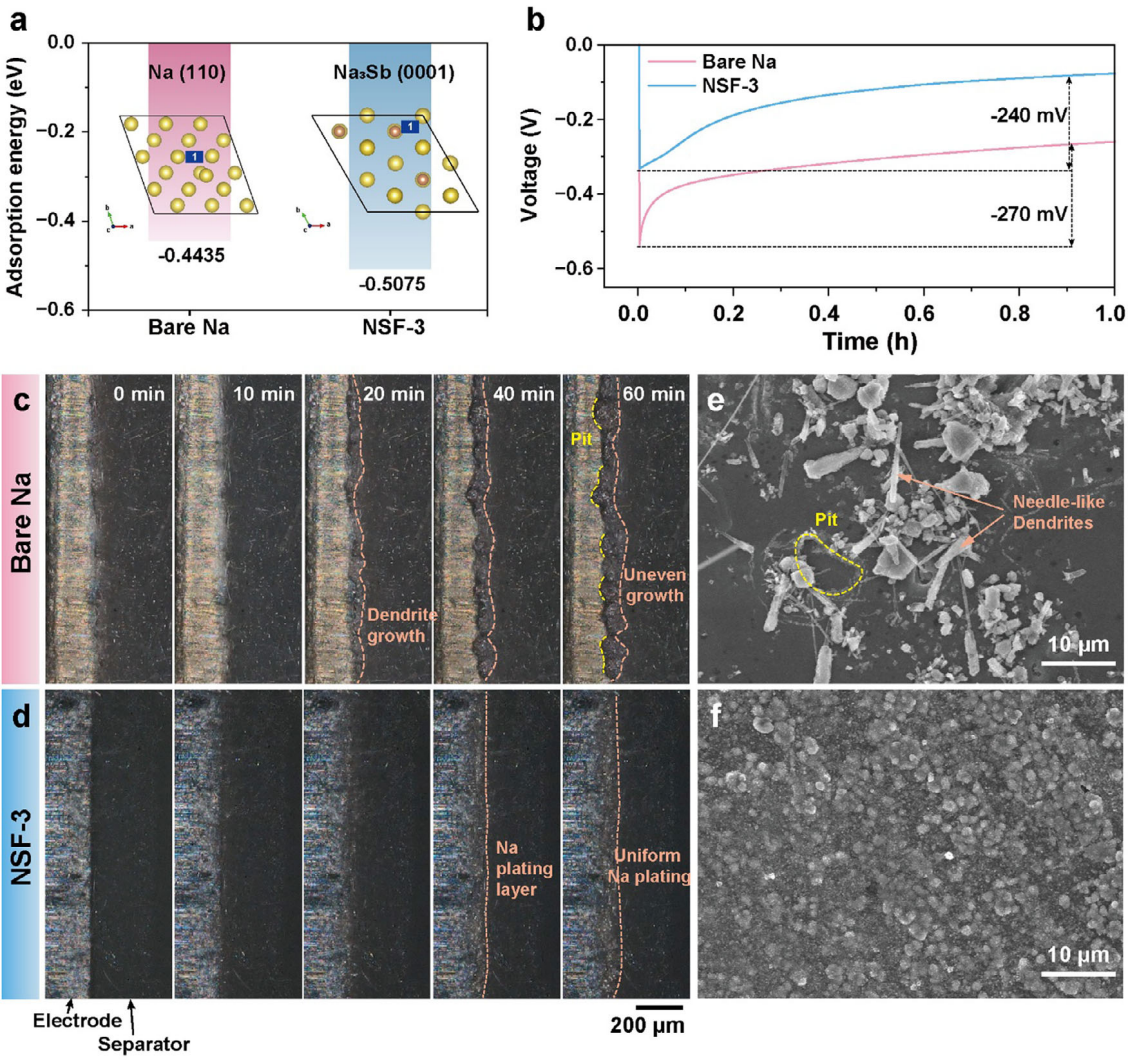
DFT analysis and visualization of Na deposition behavior on Bare Na and NSF‐3. (a) Na adsorption configurations and adsorption energies on Na(110) and Na_3_Sb(001) surfaces. (b) Initial Na plating profiles of Bare Na||Bare Na and NSF‐3||NSF‐3 symmetric cells. (c, d) In situ OM images of Na plating on Bare Na and NSF‐3, respectively. (e, f) Top‐view SEM images of Bare Na and NSF‐3, respectively, after the first Na plating.

To gain insight into the origin of the improved Na plating behavior, density functional theory (DFT) calculations were performed to evaluate the Na adsorption characteristics on metallic Na and Na_3_Sb surfaces. As shown in Figure 3a, the most energetically favorable Na adsorption sites were identified on the Na(110) and Na_3_Sb(001) surfaces, and the corresponding adsorption energies were calculated. The Na_3_Sb(001) surface exhibited a more negative adsorption energy (−0.5075 eV) than the Na(110) surface (−0.4435 eV), indicating a stronger interaction between Na atoms and the Na_3_Sb surface. The enhanced Na affinity of Na_3_Sb suggests that Na nucleation can occur more readily and uniformly on the Na_3_Sb framework, thereby providing favorable nucleation sites for subsequent Na deposition. To experimentally verify the DFT prediction, the initial Na plating behavior was investigated using symmetric cells. The voltage profile during the first plating process is shown in Figure 3b. Consistent with the calculated adsorption energies, NSF‐3 exhibited a lower nucleation overpotential than pristine Na metal, indicating a reduced nucleation barrier and more favorable Na deposition kinetics. These results suggest that the embedded Na_3_Sb framework facilitates the initial nucleation process by providing energetically favorable sites for Na adsorption and growth.

The influence of the Na_3_Sb framework on Na deposition morphology was further examined by in situ optical microscopy (OM). Optical cells were assembled using Bare Na||Bare Na and NSF‐3||NSF‐3 symmetric configurations with a glass microfiber separator, and galvanostatic plating was conducted at a current density of 0.5 mA cm^−2^ with a capacity of 0.5 mAh cm^−2^. Figure 3c presents the real‐time evolution of Na deposition on the Bare Na electrode. Dendritic Na growth became visible after approximately 20 min of plating and progressively intensified as deposition proceeded. At the end of plating (60 min), pronounced needle‐like dendrites and irregular surface features were observed. In addition, localized pit formation was detected, indicating highly non‐uniform Na deposition and stripping behavior. The ex situ SEM image obtained after the first plating cycle (Figure 3d) further confirms the coexistence of dendritic structures and pit‐like surface defects on the Bare Na electrode. In contrast, NSF‐3 exhibited markedly different deposition behavior (Figure 3e). Uniform Na deposition was observed from the early stage of plating and was maintained throughout the entire deposition process without noticeable dendrite formation. Even after 60 min of plating, the electrode surface remained smooth and homogeneous, with no evidence of dendritic protrusions or pit formation. The corresponding SEM image after the first plating cycle (Figure 3f) revealed a dense and uniform Na deposition layer with a thickness below approximately 4 µm, further confirming the highly regulated deposition behavior induced by the Na_3_Sb framework. The significantly different deposition morphologies between Bare Na and NSF‐3 can be attributed to the uniformly distributed sodiophilic Na_3_Sb framework embedded within the Na matrix. Owing to its stronger Na affinity, as evidenced by the DFT calculations, the Na_3_Sb framework provides abundant energetically favorable nucleation sites that promote homogeneous Na nucleation and spatially uniform Na‐ion flux. Such regulated deposition behavior effectively suppresses localized current concentration and dendritic growth, resulting in smooth and stable Na plating during cycling.

**FIGURE 4:**
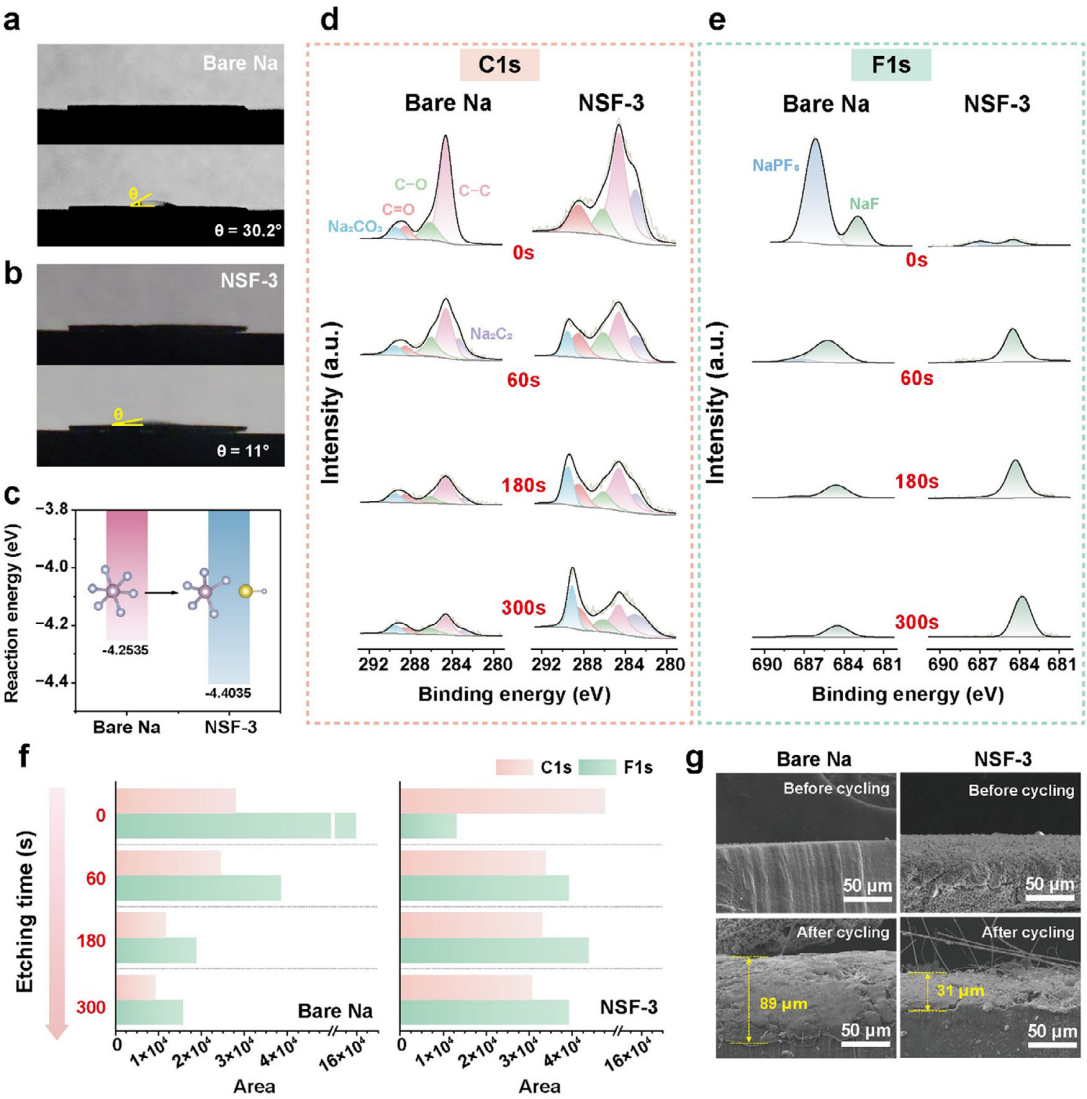
Investigation of interfacial chemistry and SEI evolution on Bare Na and NSF‐3. (a, b) Electrolyte contact angle measurements. (c) Reaction energy for the decomposition of PF_6_ into PF_5_ and NaF, as calculated by DFT. The decomposition process is illustrated in the figure using VESTA. (d, e) C1s and F1s XPS depth‐profile analysis for bare Na and NSF‐3 after 50 cycles. (f) Changes in the intensity of characteristic XPS peaks for bare Na as a function of etching time. (g) Cross‐sectional SEM images of bare Na and NSF‐3 before and after cycling.

The interfacial characteristics of Bare Na and NSF‐3 were investigated through electrolyte wettability measurements. As shown in Figure 4a,b, Bare Na exhibited a contact angle of 30.2°, whereas NSF‐3 showed a significantly lower contact angle of 11.0°. The reduced contact angle indicates enhanced electrolyte affinity and improved wettability on the NSF‐3 surface. Such favorable electrolyte/electrode interactions are expected to facilitate homogeneous electrolyte distribution and promote the formation of a more uniform interphase during the initial stages of cycling [16, 17]. To further examine the interfacial reaction behavior, DFT calculations were performed to evaluate the reaction energy of reductive decomposition of PF_6_^−^ on Bare Na and NSF‐3 surfaces. As shown in Figure 4c, the calculated reaction energy on NSF‐3 (−4.4035 eV) was lower than that on Bare Na (−4.2535 eV), indicating that PF_6_^−^ decomposition is thermodynamically more favorable on the NSF‐3 surface. Because PF_6_^−^ decomposition is closely associated with the formation of inorganic NaF species, the DFT results suggest that NSF‐3 may promote the formation of a NaF‐rich interphase during cycling. Such NaF‐rich SEI layers are widely recognized for their high mechanical robustness and electronic insulating properties, which are beneficial for suppressing dendrite growth and regulating Na deposition behavior. To verify the interfacial chemistry after cycling, XPS depth‐profile analyses were performed on Bare Na and NSF‐3 electrodes after 50 cycles. Figure 4d and Figure S15 present the C 1s and O 1s/Sb3d spectra collected at different etching times. For both electrodes, characteristic peaks corresponding to C–C, C–O, C═O, and C–F species were observed near the surface, indicating the presence of organic electrolyte decomposition products. These organic components originate from the decomposition products of a typical carbonate‐based electrolyte [35, 36, 37]. However, the evolution of the peak intensities during depth profiling revealed distinct differences between the two electrodes. For Bare Na, the intensities of the carbon‐containing species rapidly decreased with increasing etching time and nearly disappeared after prolonged etching, suggesting a relatively non‐uniform distribution of SEI components throughout the interphase. In contrast, NSF‐3 exhibited a gradual decrease in organic species accompanied by an increasing contribution from inorganic components such as Na_2_CO_3_ and Na_2_O with increasing etching depth. These observations indicate the formation of a compositionally graded SEI in which the inorganic content becomes increasingly dominant toward the inner region.

To further elucidate the fluorine‐containing species within the SEI, F 1s spectra were analyzed and are shown in Figure 4e. The evolution of the relative intensities of PF_6_^−^‐derived species and NaF as a function of etching time is summarized in Figure 4f. For Bare Na, a strong PF_6_^−^‐related signal and a relatively weak NaF signal were observed, while the NaF intensity gradually decreased with increasing etching depth. This suggests that a significant fraction of PF_6_^−^‐derived species remained within the interphase without complete conversion into stable inorganic products. In contrast, NSF‐3 exhibited a much weaker PF_6_^−^ signal and a progressively increasing NaF contribution with increasing etching time. These results indicate more effective PF_6_^−^ decomposition on NSF‐3, leading to the formation of a NaF‐rich inorganic interphase. Combined with the C 1s and O 1s/Sb3d analyses, the XPS depth‐profile results demonstrate that NSF‐3 forms a well‐organized multilayered SEI composed of an outer organic‐rich region and an inner inorganic‐rich region containing NaF, Na_2_CO_3_, and Na_2_O [38]. As shown in Figure S15b, no distinct Sb 3d_5/2_ signal corresponding to metallic Sb was observed on the NSF‐3 surface before Ar^+^ etching after 50 cycles. In contrast, a peak corresponding to metallic Sb appeared at approximately 528 eV after Ar^+^ etching. This signal may originate from the preferential removal of Na from the Na_3_Sb phase during Ar^+^ sputtering, which increases the relative Sb content at the etched surface and consequently leads to the appearance of the metallic Sb signal.

The differences in interphase stability were further reflected in the electrode morphology after cycling. As shown in Figure 4g, Bare Na exhibited a substantial electrode thickness increase of approximately 89 µm after cycling, whereas NSF‐3 showed a significantly smaller increase of only 31 µm. The pronounced volume expansion observed for Bare Na is likely associated with heterogeneous Na deposition, continuous interphase reconstruction, and the accumulation of electrically isolated (“dead”) Na during cycling. In contrast, the considerably smaller thickness change observed for NSF‐3 suggests the formation of a mechanically robust and stable interphase. The NaF‐rich inorganic SEI generated on NSF‐3 can effectively accommodate interfacial stress and regulate Na deposition, thereby mitigating electrode swelling and suppressing dendrite growth during prolonged cycling [39, 40, 41, 42].

**FIGURE 5:**
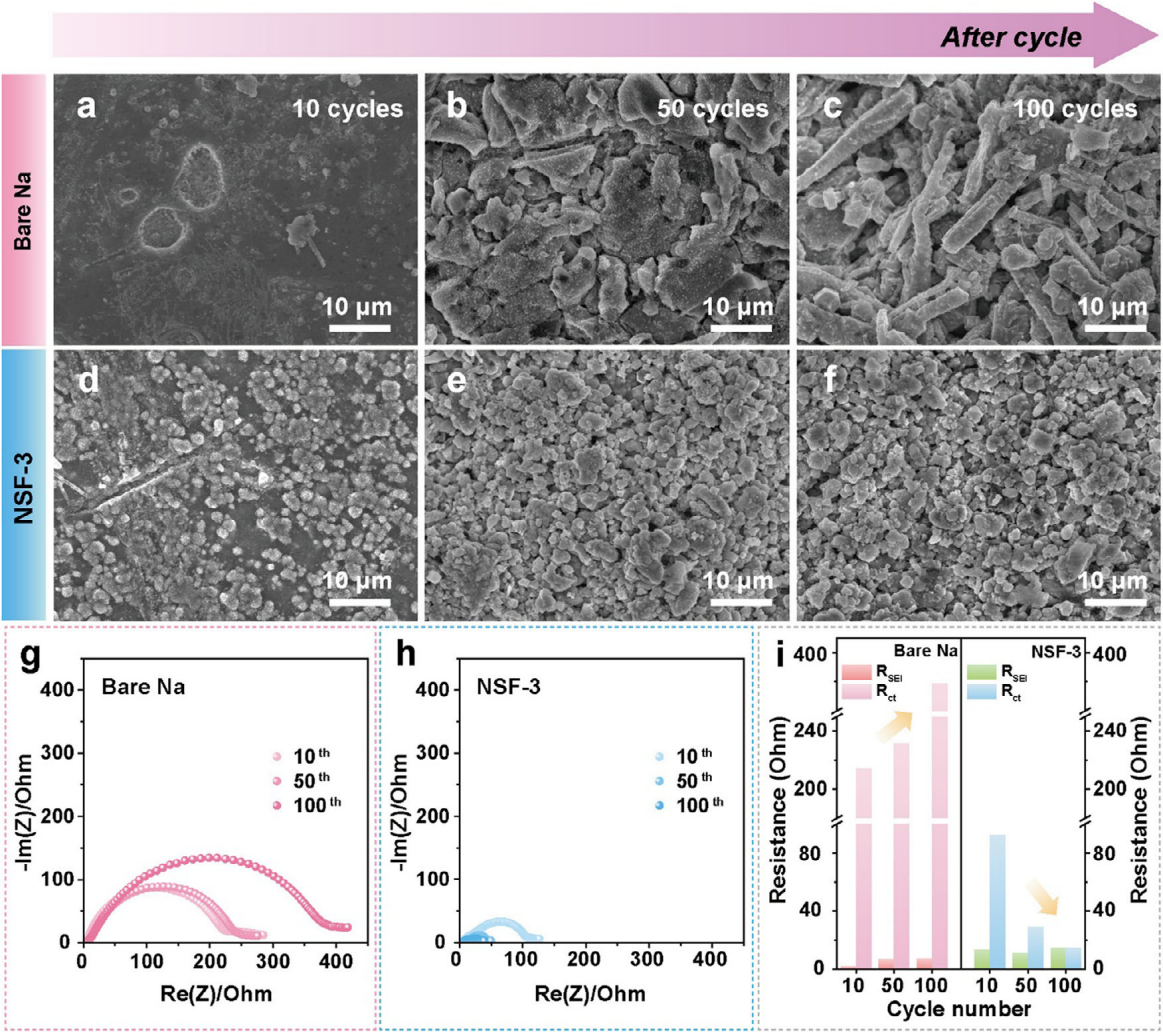
Morphological and electrochemical evolution of Bare Na and NSF‐3 during cycling. (a–c) Top‐view SEM images of Bare Na and (d–f) NSF‐3 after 10, 50, and 100 cycles at 0.5 mA cm^−2^ and 0.5 mAh cm^−2^. (g, h) Nyquist plots of Bare Na and NSF‐3 at different cycle numbers. (i) Corresponding R_SEI_ and R_ct_ values obtained from EIS fitting.

To investigate the long‐term interfacial evolution during cycling, the surface morphologies of Bare Na and NSF‐3 were examined after 10, 50, and 100 cycles, and the corresponding SEM images are shown in Figure 5a–f. For Bare Na, repeated Na plating/stripping resulted in progressively roughened surface morphologies accompanied by the formation of porous interfacial structures. As cycling proceeded, numerous rod‐like features with dimensions exceeding approximately 15 µm were observed (Figure 5c), which can be attributed to accumulated dead Na generated by non‐uniform Na deposition and incomplete stripping. The continuous generation of dead Na and heterogeneous interphase evolution gradually increased the surface roughness and porosity of the electrode, indicating deterioration of the electrode/electrolyte interface during cycling. In contrast, NSF‐3 maintained a relatively smooth and compact surface morphology throughout cycling (Figure 5d–f). Even after 100 cycles, a dense interfacial layer with a thickness below approximately 10 µm was observed without obvious dendritic features or severe surface roughening. The markedly different morphological evolution compared with Bare Na suggests that the Na_3_Sb framework effectively regulates Na deposition behavior and preserves interfacial stability during prolonged cycling [11]. These observations are consistent with the in situ OM and XPS results discussed above, which demonstrated uniform Na deposition and the formation of a stable NaF‐rich interphase on NSF‐3.

To quantitatively evaluate the interfacial evolution during cycling, electrochemical impedance spectroscopy (EIS) measurements were performed after 10, 50, and 100 cycles. The Nyquist plots are shown in Figure 5g,h, while the corresponding equivalent circuit model and fitting parameters are provided in Figure S16 and Table S3, respectively. In the fitted circuit, the high‐frequency semicircle corresponds to the SEI resistance (R_SEI_), whereas the medium‐frequency semicircle represents the charge‐transfer resistance (R_ct_) at the electrode/electrolyte interface. As shown in Figure 5g, Bare Na exhibited a continuous increase in both R_SEI_ and R_ct_ with increasing cycle number. The increasing R_ct_ indicates progressively hindered charge‐transfer kinetics, which can be associated with the accumulation of dead Na and the development of a heterogeneous interfacial structure. Although the measured R_SEI_ remained relatively low, the simultaneous increase in R_ct_ suggests continuous interphase reconstruction and unstable Na deposition behavior throughout cycling. The accumulation of dead Na generated during this process blocks ion transport pathways, further inhibiting charge transfer at the interface [34, 43, 44]. In contrast, NSF‐3 exhibited markedly different impedance evolution (Figure 5h and Figure S17). The R_SEI_ remained nearly unchanged during cycling, indicating the formation of a stable interphase with minimal structural disruption. More importantly, the R_ct_ gradually decreased with cycling, suggesting improved interfacial charge‐transfer kinetics as the electrode/electrolyte interface became progressively stabilized. The stable NaF‐rich SEI identified by XPS analysis likely facilitates uniform ion transport while suppressing continuous electrolyte decomposition and interphase reformation. The evolution of R_SEI_ and R_ct_ is summarized in Figure 5i. Compared with Bare Na, NSF‐3 maintained significantly lower and continuously decreasing R_ct_ values while preserving a stable RSEI throughout cycling. These results demonstrate that the Na_3_Sb framework enables the formation of a robust and stable electrode/electrolyte interface, thereby facilitating efficient charge transfer and sustaining uniform Na plating/stripping during prolonged cycling.

**FIGURE 6:**
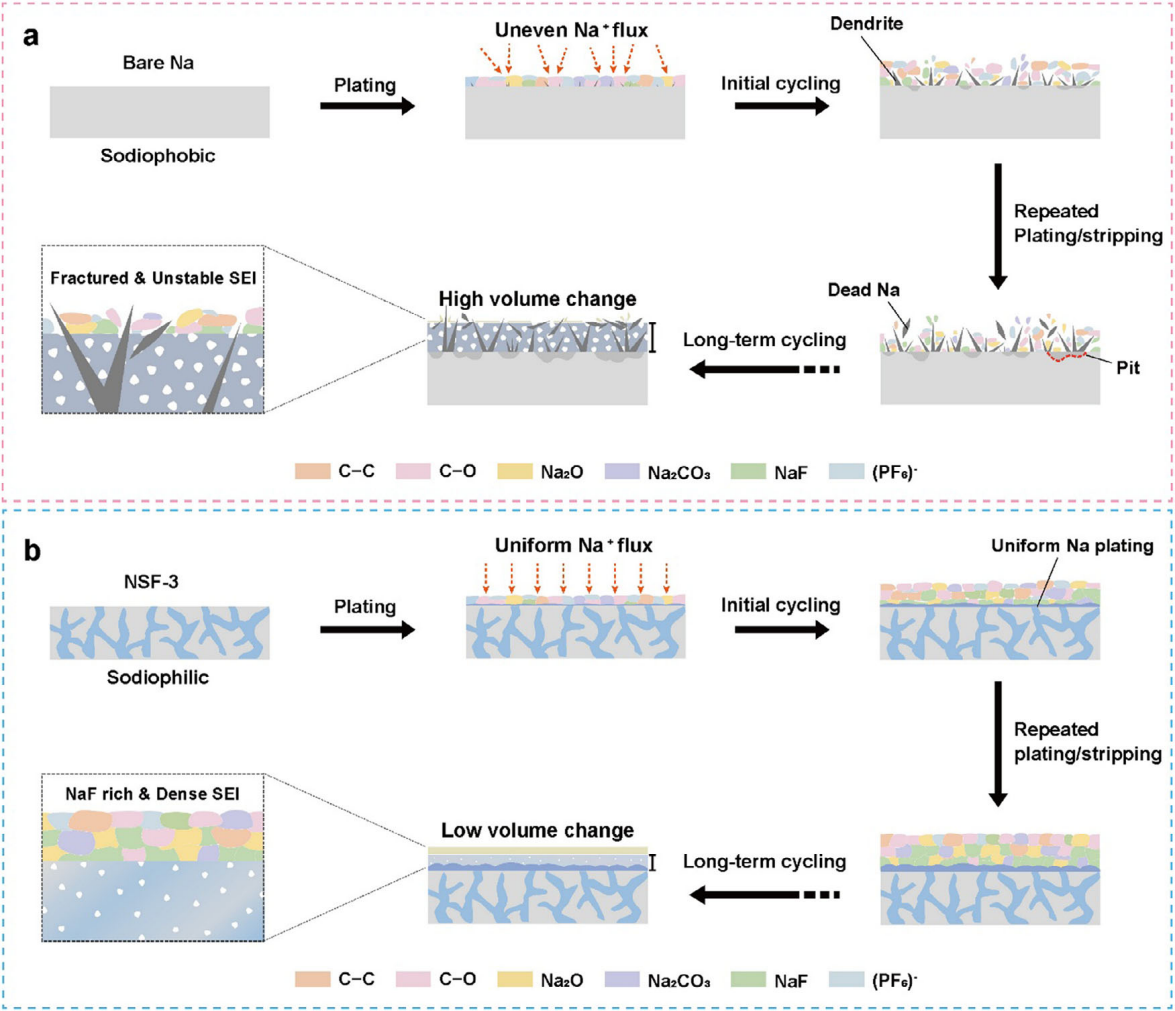
Schematic illustration of SEI formation and interfacial stabilization mechanisms of (a) Bare Na and (b)NSF‐3.

Figure 6a,b summarizes the interfacial evolution mechanisms of Bare Na and NSF‐3 during repeated Na plating/stripping. For Bare Na, Na deposition preferentially occurs at localized regions owing to the limited number of favorable nucleation sites. The resulting non‐uniform Na‐ion flux induces localized current concentration and promotes dendritic Na growth. As cycling proceeds, the continuously growing dendrites repeatedly fracture the SEI layer, exposing fresh Na surfaces to the electrolyte and triggering further electrolyte decomposition. This continuous interphase reconstruction leads to the accumulation of dead Na, the formation of porous surface structures and deep pits, and substantial volume fluctuation. Consequently, the electrode/electrolyte interface becomes increasingly unstable, eventually resulting in severe polarization and premature cell failure. In contrast, the uniformly distributed Na_3_Sb framework embedded within the Na matrix provides abundant sodiophilic nucleation sites, as supported by the DFT calculations and nucleation overpotential analyses. The enhanced Na affinity of Na_3_Sb promotes homogeneous Na nucleation and regulates Na‐ion flux across the electrode surface, thereby suppressing localized current concentration and dendritic growth. In addition, the NSF‐3 surface facilitates PF_6_^−^ decomposition, leading to the preferential formation of a NaF‐rich inorganic interphase. The mechanically robust NaF‐rich SEI effectively stabilizes the electrode/electrolyte interface, suppresses continuous electrolyte decomposition, and accommodates interfacial stress during cycling. As a result, NSF‐3 maintains a dense and stable interphase, enabling highly reversible Na plating/stripping and long‐term cycling stability.

To further evaluate the practical applicability of the proposed anode, full‐cell tests were conducted using Na_0.9_Ni_0.45_Mn_0.55_O_2_ (NNMO) as the cathode with a cathode loading of 10.41 mg cm^−2^ and either Bare Na or NSF‐3 as the anode. The crystal structure and morphology of NNMO were confirmed by XRD and SEM analyses (Figure S18), which revealed the formation of a phase‐pure layered oxide with a uniform particle morphology. The cycling performance of the full cells is presented in Figure S19. The NNMO||NSF‐3 full cell delivered stable cycling performance for more than 150 cycles at a current density of 200 mA g^−^^1^ (Figure S19a), demonstrating the compatibility of the Na_3_Sb‐modified anode with practical cathode materials. Representative galvanostatic charge‐discharge profiles are shown in Figure S19b–d. The discharge plateau near 2.5 V corresponds to the Mn^4+^/Mn^3+^ redox reaction of NNMO [45]. Notably, the Bare Na full cell exhibited noticeable voltage fluctuations during prolonged cycling, particularly in the charging region above 4.0 V, which is commonly associated with interfacial instability and dendrite‐induced micro‐short‐circuit behavior [46, 47, 48, 49]. In contrast, the NNMO||NSF‐3 full cell maintained stable voltage profiles without abnormal voltage noise throughout cycling. Consistent voltage profiles were observed even after 5, 10, 50, 100, and 150 cycles, indicating highly reversible electrochemical behavior.

Beyond the NNMO‐based full‐cell evaluation, the electrochemical performance of NSF‐3 was also examined in a different cathode system using Na_3_V_2_(PO_4_)_3_ (NVP) with a cathode loading of approximately 5.22 mg cm^−2^. For comparison, NVP was paired with either Bare Na or NSF‐3, and the full cells were evaluated within a voltage range of 2.0–3.8 V (Figure S20a–e). At a high current density of 400 mA g^−1^, the NVP||NSF‐3 full cell maintained a high discharge capacity of approximately 102.7 mAh g^−1^ after 320 cycles. In contrast, the NVP||Bare Na full cell exhibited a noticeable capacity decrease during the initial ∼20 cycles before gradually stabilizing, delivering approximately 94.7 mAh g^−1^ after 320 cycles. Notably, the NVP||NSF‐3 full cell maintained stable cycling behavior without an obvious capacity decay from the initial cycles, indicating that the stable interfacial characteristics enabled by the Na_3_Sb framework were effectively maintained during prolonged cycling. In addition, the rate capability of the NVP||NSF‐3 full cell was evaluated, and a discharge capacity of approximately 94.7 mAh g^−1^ was maintained even at a high current density of 300 mA g^−1^. To examine the feasibility of NSF‐3 for practical SMB applications, a single‐layer pouch cell was fabricated using an NVP cathode with a cathode loading of approximately 1.88 mg cm^−2^ and an NSF‐3 anode (Figure S20f–h). The pouch cell was evaluated within the same voltage range of 2.0–3.8 V and exhibited stable cycling behavior at a current density of 100 mA g^−1^, maintaining a discharge capacity of approximately 93.2 mAh g^−1^ after 50 cycles. These results demonstrate that NSF‐3 maintains stable electrochemical performance in a single‐layer pouch cell configuration, supporting its applicability to SMB systems.

Collectively, these results demonstrate that the superior electrochemical performance of NSF‐3 originates from the synergistic effects of the embedded Na_3_Sb framework on both Na deposition behavior and interfacial chemistry. The uniformly distributed sodiophilic Na_3_Sb phase provides energetically favorable nucleation sites, lowers the Na nucleation barrier, and promotes homogeneous Na‐ion flux across the electrode surface. As a result, localized current concentration is effectively mitigated, leading to highly uniform Na deposition and the suppression of dendritic growth throughout repeated cycling. Simultaneously, the enhanced interfacial reactivity of NSF‐3 facilitates PF_6_^−^ decomposition and promotes the formation of a mechanically robust NaF‐rich SEI. This stable inorganic‐rich interphase effectively minimizes continuous electrolyte decomposition, suppresses dead Na accumulation, and preserves interfacial integrity during prolonged Na plating/stripping processes. More importantly, the advantages observed in symmetric cells were successfully translated to practical full‐cell configurations employing different cathode materials and were further demonstrated in a single‐layer pouch cell, confirming the stable electrochemical performance of NSF‐3 across different cathode systems and cell formats. Therefore, this work highlights the incorporation of a sodiophilic Na_3_Sb framework into Na metal as an effective strategy for simultaneously regulating Na nucleation, deposition, and interphase evolution, thereby addressing the critical challenges associated with Na metal anodes. The proposed NSF‐3 electrode provides a scalable and practical pathway toward the realization of high‐performance, dendrite‐free, and long‐life SMBs.

## 3 Conclusions

In this work, we developed a Na metal composite anode containing a uniformly distributed Na_3_Sb framework embedded within a Na matrix through a simple and scalable metallurgical process. Owing to its enhanced sodiophilicity, the Na_3_Sb framework provides abundant energetically favorable nucleation sites for Na deposition, thereby lowering the nucleation barrier and promoting homogeneous Na‐ion flux across the electrode surface. In addition, the presence of Na_3_Sb facilitates PF_6_^−^ decomposition and promotes the formation of a mechanically robust NaF‐rich SEI, which effectively stabilizes the electrode/electrolyte interface during repeated Na plating/stripping. As a result, the NSF‐3 anode exhibited highly uniform Na deposition behavior, suppressed dendrite growth, reduced charge‐transfer resistance, and significantly improved interfacial stability compared with pristine Na metal. The NSF‐3||NSF‐3 symmetric cell delivered stable cycling performance for over 1000 h with low polarization under a current density of 0.5 mA cm^−2^ and a capacity of 0.5 mAh cm^−2^. Furthermore, the NNMO||NSF‐3 full cell demonstrated stable cycling performance for more than 150 cycles at a current density of 200 mA g^−1^, confirming the practical applicability of the proposed anode design. Overall, this study demonstrates that incorporating a sodiophilic Na_3_Sb framework into Na metal is an effective strategy for simultaneously regulating Na nucleation, deposition behavior, and interphase evolution. The synergistic combination of uniform Na deposition and stable NaF‐rich interphase formation provides a promising pathway toward the development of high‐performance, dendrite‐free, and long‐life SMBs.

## Author Contributions

**So Hyun Lee**: methodology, conceptualization, investigation, writing – original draft, data curation, visualization. **Dawon Baek**: methodology, data curation, validation, investigation. **Inkyung Kim**: methodology, software, validation. **Sang Jun Lee**: methodology, formal analysis. **Ho Jin Ma**: methodology, data curation, supervision. **Tae Hoon Hwang**: supervision, funding acquisition, project administration, resources. **San Moon**: supervision, writing – original draft, validation, investigation. **Joo‐Hyung Kim**: methodology, conceptualization, visualization, writing – original draft, supervision, funding acquisition.

## Conflicts of Interest

The authors declare no conflicts of interest.

## Supporting information




**Supporting File**: advs77935‐sup‐0001‐SuppMat.docx.

## Data Availability

The data that supports the findings of this study are available in the supplementary material of this article.
