# Supplementary material for: Sodiophilic Na3Sb Framework‐Regulated Sodium Deposition and Interphase Evolution for Dendrite‐Free and Long‐Life Sodium Metal Batteries
Source: Adv Sci (Weinh). 2026 Sep 27:e77935. Online ahead of print. doi: 10.1002/advs.77935 (PMC13616310; doi:10.1002/advs.77935)
Supplement: Supplementary file 1 [file ADVS-9999-e77935-s001.docx]

Supporting Information

Sodiophilic Na_3_Sb Framework-Regulated Sodium Deposition and Interphase Evolution for Dendrite-Free and Long-Life Sodium Metal Batteries

**1. Experimental section**

***1.1. Synthesis of NSF-3***

All processes were conducted in an argon (Ar)-filled glove box, and NSF-3 was prepared via a simple metallurgical process. A Na metal cube (99.9%, Sigma Aldrich) was completely melted in an alumina crucible using a 300°C hotplate. After that Sb powder (99.8%, Sigma Aldrich) was then slowly added at a Na:Sb ratio of 3:0.3 (molar ratio, atomic percent = 9.1 at%), and the mixture was stirred for 20 minutes using a stainless steel spatula before being cooled to room temperature(25°C) on an alumina plate. Subsequently, NSF-3 with a thickness of approximately 250 μm was fabricated using a mechanical rolling process.

***1.2. Material characterizations***

All sample analyses were performed inside an Ar-filled glove box. X-ray diffraction (XRD) was performed using a D8 Advance A25 Plus (Bruker) with Cu Kα radiation (λ = 1.5418 Å). Scanning electron microscopy (SEM) and energy-dispersive spectroscopy (EDS) mapping of samples were observed by a JSM-7610F (JEOL) at acceleration voltages of 10 kV and 15 kV, respectively. X-ray photoelectron spectroscopy (XPS) were conducted on a NEXSA (ThermoFisher) spectrometer with Al Kα radiation. In situ optical microscopy (OM) was performed using a DSX1000 (OLYMPUS). After the cycling was completed, all electrodes were disassembled, washed several times with propylene carbonate (PC) solvent, and vacuum-dried at room temperature for 30 minutes.

***1.3 Electrochemical measurement***

The NSF-3||NSF-3 and Bare Na||Bare Na symmetric cells, assembled using CR2032 coin cells, were evaluated at room temperature using an Integrated Constant Environmental Test Chamber (MIHW-200-160CH-B, NEWARE). The evaluation used an electrolyte consisting of a 6:4(volume per volume, v/v) mixture of propylene carbonate (PC) and ethyl methyl carbonate (EMC), combined with 1 M NaPF_6_ and 5 wt% fluoroethylene carbonate (FEC) as an additive, and glass microfiber filters (GF/D, Whatman) was used as the separator. Linear sweep voltammetry (LSV) was measured in the range of -0.3 V to 0.3 V using an Automatic Battery Cycler (WBCS300L, WonATech), and EIS measurements before and after cycling were performed in the frequency range of 1 MHz to 1 mHz using an SP-300 (BioLogic). The Na_0.9_Ni_0.45_Mn_0.55_O_2_ and Na_3_V_2_(PO4)_3_ (NVP)cathode used in the full-cell test, and the binder was prepared by mixing Poly(vinylidene fluoride)(PVdF, Typolymer) with anhydrous1-Methyl-2-pyrrolidone (NMP, Sigma Aldrich) in a 1:1 ratio. The slurry was prepared with an active material:super P:binder ratio of 8:1:1. It was then cast onto aluminum(Al) foil, dried overnight in a vacuum oven at 80°C, and punched into 12 mm discs. Full cells employing either Bare Na or NSF-3 as the anode were assembled in CR2032-type coin cells. For the single-layer pouch cell, a 3 × 3 cm^2^ NVP cathode cast on Al foil, a 3.2 × 3.2 cm^2^ NSF-3 anode, and a 3.5 × 3.5 cm^2^ GF/D separator were used for cell assembly. The SP-300 (BioLogic) was used for the electrochemical testing of the in situ OM, and the electrodes were cut into 10 mm × 10 mm squares.

***1.4. DFT calculations***

All spin-polarized density functional theory (DFT) calculations were performed using the Vienna Ab-initio Simulation Package (VASP). [1] The interactions between core and valence electrons were described using the projector augmented wave (PAW) method. [2] Exchange–correlation effects were treated within the generalized gradient approximation (GGA) using the Perdew–Burke–Ernzerhof (PBE) functional. [3] The electronic wave functions were expanded in a plane-wave basis set with a kinetic energy cutoff of 500 eV.

To investigate adsorption and interfacial reaction behaviors, two surface models were constructed: a Na(110) surface and a Na₃Sb(0001) surface. To minimize lateral interactions between periodically repeated adsorbates, the lateral dimensions of the surface supercells were chosen to be approximately 10 Å, corresponding to a 3 × 3 supercell for Na(110) and a 2 × 2 supercell for Na₃Sb(0001). The Na(110) and Na₃Sb(0001) slabs consisted of four and five atomic layers, respectively. A vacuum region of approximately 10 Å was introduced along the surface-normal direction to eliminate spurious interactions between periodic slab images.

Brillouin zone integrations were performed using Monkhorst–Pack k-point meshes [4] of 2 x 2 x 1 for both the Na(110) and Na₃Sb(0001) surfaces. The adsorption energy of a Na atom and the dissociation energy of PF₆ were calculated on the hollow site of Na(110) and the Sb-top site of Na₃Sb(0001) according to Equations (1) and (2), respectively.

$E_{\text{ads Na}}\text{ }= \text{E}_{\text{Na/Slab}}-(\text{E}_{\text{Na}}+\text{E}_{\text{Na}})$	(1)

The dissociation energy was evaluated for the Na-incuded PF_6_ decomposition reaction on the surface: $\text{PF}_{\text{6}}+\text{Na}+\text{slab}\to\text{PF}_{\text{5}}+\text{Na-F}/\text{slab}$

$\text{E}_{\text{diss}\text{o}_{\text{PF}_{\text{6}}}}=\left( \text{E}_{\text{PF}_{\text{5}}}+\text{E}_{\text{Na-F/slab}} \right)-(\text{E}_{\text{Na}}+\text{E}_{\text{PF}_{\text{6}}}\text{+}\text{E}_{\text{slab}})$	(2)

In these equations, E_slab_ is the total energy of the pristine slab, and E_Na_ is the reference energy of a Na atom obtained from bulk body-centered cubic (BCC) metallic Na. For Equation (1), E_Na/slab_ is the total energy of the slab with an adsorbed Na atom. For Equation (2), E_PF5_ and E_PF6_ denote the total energies of isolated PF_5_ and PF_6_ species, respectively. E_Na-F/slab_ represents the total energy of the slab with adsorbed Na and F atoms.

**Figure S1.** Photographs of electrode showing an increase in Sb content. From left to right, they show Bare Na, NSF-1(Na:Sb = 3:0.1), NSF-2(3:0.2), NSF-3(3:0.3), and NSF-4(3:0.4) electrodes, respectively

**Figure S2.** (a) XRD pattern and (b)SEM image of Sb powder

**Figure S3.** (a) SEM images of Bare Na with the corresponding EDS mapping of element (b) Na

**Figure S4.** BSE image of NSF-3

**Figure S5.** Comparison of Na plating/stripping behavior on Bare Na, NSF-1, NSF-2, NSF-3, and NSF-4. Time-voltage profiles measured at a current density of 1 mA cm^-2^ with a capacity of 1 mAh cm^-2^ (a) Overall voltage profile up to 300 h. (b-c) These zoomed-in voltage profile at different cycling states.

**Figure S6.** (a) Nucleation overpotential profile of Bare Na, Na, NSF-1, NSF-2, NSF-3 and NSF-4. (b) A plot comparing the nucleation overpotential values for each electrode.

**Figure S7.** Electrochemical stripping process and structural analysis for removing excess Na from the NSF-3. (a) Time-voltage profile of the NSF-3 measured at a current density of 1mA cm^-2^ and a capacity of 29 mAh cm^-2^ (b)XRD pattern of the NSF-3 after electrochemical stripping. (c,d) Low- and high-magnification SEM images of NSF-3 after deep-stripping to remove metallic Na.

**Figure S8**. Surface XPS spectra of the O 1s and Sb 3d regions for NSF-3.

**Figure S9**. Time-voltage profiles of Bare Na and NSF-3. Short-circuit behavior observed under (a) 1 mA cm^-2^, 1 mAh cm^-2^, (b) 0.5 mA cm^-2^, 0.5 mAh cm^-2^. Long-term Na plating/stripping performance at (a) 1 mA cm^-2^, 1 mAh cm^-2^ for 300 h and (b) 0.5 mA cm^-2^, 0.5 mAh cm^-2^ for 1000 h.

**Figure S10.** Time-voltage profiles at 1 mA cm^-2^, 1 mAh cm^-2^ of Bare Na and NSF-3. (a)The initial cycle and (b) the middle cycle

**Figure S11**. Time-voltage profiles at 0.5 mA cm^-2^, 1 mAh cm^-2^ of Bare Na and NSF-3.

**Figure S12.** Electrochemical kinetics of Bare Na and NSF-3 symmetric cells in the initial state. (a) LSV curves and corresponding slopes. (b) Tafel plot and exchange current densities (j0) calculated from the Tafel plot.

**Figure S13.** LSV and Tafel analyses of Bare Na and NSF-3 symmetric cells after cycling to evaluate the evolution of interfacial charge-transfer kinetics. LSV curves after (a) 5 and (b) 10 cycles, with the corresponding slope values indicated in each panel. Tafel plot after (c) 5 and (d) 10 cycles, with the exchange current densities (j_0_) calculated from the Tafel plot indicated in each panel.

**Figure S14.** Cyclic voltammetry(CV) curves of Bare Na and NSF-3 measured over a voltage range of -0.3 to 0.3 V at different scan rates of (a) 0.1, (b) 0.5, (c) 2, and (d) 5 mV s^-1^.

**Figure S15**. XPS depth profiles of cycled Bare Na and NSF-3 electrodes in symmetric cells after 50 cycles. O1s/Sb3d spectra of (a) Bare Na and (b) NSF-3.

**Figure S16.** Equivalent circuit model for EIS analysis

**Figure S17.** Enlarged Nyquist plot of NSF-3.

**Figure S18.** (a) XRD pattern and (b) SEM image of Na_0.9_Ni_0.55_Mn_0.45_O_2_.

**Figure S19.** (a) Cycling performance of the full cells with a Bare Na and NSF-3 anode and a of Na_0.9_Ni_0.55_Mn_0.45_O_2_ cathode at 200 mA g^-1^. Corresponding GCD curves at the (b) 1^st^ and (c) 52^nd^ cycle. (d) GCD curves of the of Na_0.9_Ni_0.55_Mn_0.45_O_2_||NSF-3 full cell from the 5^th^ to the 150^th^ cycle.

**Figure S20.** Electrochemical performance of Bare Na and NSF-3 full cells using Na_3_V_2_(PO_4_)_3_ (NVP) as the cathode. (a) Schematic illustration of the CR2032 coin-cell configuration. (b) Long-term cycling performance of NVP||Bare Na and NVP||NSF-3 full cells at a current density of 400 mA g^-1^. Corresponding galvanostatic charge-discharge profiles of (c) Bare Na and (d) NSF-3 full cells after cycling. (e) Rate performance of the NSF-3 full cell. (f) Schematic illustration of the single-layer NVP||NSF-3 pouch cell. (g) Cycling performance of the NVP||NSF-3 pouch cell at a current density of 100 mA g^-1^. (h) Corresponding galvanostatic charge-discharge profiles of the pouch cell.

|  | **Na** | **Na_3_Sb** |
| --- | --- | --- |
| **Melting temperature(°C)** | 97.8 °C | ~857°C |
| **Elastic modulus (GPa)** | 3.9 [4] | 27.86 [5] |
| **Shear modulus G_VRH_ (GPa)** | 3 | 11 |
| **Bulk modulus K_VRH_ (GPa)** | 8 | 14 |
| **Electronic conductivity (mS m^-1^)** | 9.18×10^-2^ [6] | 9.6×10^-3^ [6] |

**Table S1.** Comparison of the thermal, mechanical, and electronic properties of Na and Na_3_Sb.

**Table S2.** Comparison of voltage polarization values for Bare Na and NSF-X (X = 1, 2, 3, 4) electrodes after cycling.

|  | **Sample name** | | | | |
| --- | --- | --- | --- | --- | --- |
| **Cycle number** | **Bare Na** | **NSF-1** | **NSF-2** | **NSF-3** | **NSF-4** |
| **30^th^** | 290 [mV] | 135 | 123 | 132 | 80 |
| **150^th^** | 47 | 61 | 81 | 72 | 73 |
| **300^th^** | - | 98 | 81 | 71 | 100 |

**Table S3.** The EIS fitting results of the Bare Na and NSF-3 electrode recorded at different cycles

|  | **Bare Na** | | | **NSF-3** | | |
| --- | --- | --- | --- | --- | --- | --- |
|  | **10** | **50** | **100** | **10** | **50** | **100** |
| **R_SEI_** | 2.17 | 6.84 | 7.46 | 13.61 | 10.97 | 14.80 |
| **R_ct_** | 214.38 | 231.74 | 387.85 | 92.26 | 29.42 | 14.71 |

**References**

[1] G. Kresse, J. Furthmüller, Efficiency of ab-initio total energy calculations for metals and semiconductors using a plane-wave basis set, Computational Materials Science 6 (1996) 15–50. https://doi.org/https://doi.org/10.1016/0927-0256(96)00008-0.

[2] P.E. Blöchl, Projector augmented-wave method, Physical review B 50 (1994) 17953.

[3] J.P. Perdew, K. Burke, M. Ernzerhof, Generalized gradient approximation made simple, Physical review letters 77 (1996) 3865.

[4] L. Bodenes, A. Darwiche, L. Monconduit, and H. Martinez, “The Solid Electrolyte Interphase a Key Parameter of the High Performance of Sb in Sodium-Ion Batteries: Comparative X-Ray Photoelectron Spectroscopy Study of Sb/Na-Ion and Sb/Li-Ion Batteries,” Journal of Power Sources 273 (2015): 14–24. https://doi.org/10.1016/j.jpowsour.2014.09.037

[5] C. D. Wagner, A. V. Naumkin, A. Kraut-Vass, J. W. Allison, C. J. Powell, and J. R. Rumble Jr., NIST Standard Reference Database 20, Version 3.4 (Web Version), National Institute of Standards and Technology, Gaithersburg, MD (2003)

[6] A. Livshits and M. Polak, “An X-Ray Photoelectron-Spectroscopy Study of Naβ-Alumina Surfaces,” Solid State Ionics 6, no. 4 (1982): 311–318. https://doi.org/10.1016/0167-2738(82)90015-7

[7] G. Greczynski and L. Hultman, “Towards Reliable X-Ray Photoelectron Spectroscopy: Sputter-Damage Effects in Transition Metal Borides, Carbides, Nitrides, and Oxides,” Applied Surface Science 542 (2021): 148599. https://doi.org/10.1016/j.apsusc.2020.148599

[8] Y.-C. Lin, Y.-Y. Chen, B.-Y. Yu, W.-C. Lin, C.-H. Kuo, and J.-J. Shyue, “Sputter-Induced Chemical Transformation in Oxoanions by Combination of C60+ and Ar+ Ion Beams Analyzed with X-Ray Photoelectron Spectrometry,” Analyst 134, no. 5 (2009): 945–951. https://doi.org/10.1039/B814729A

[9] C. D. Fincher, Y. Zhang, G. M. Pharr, and M. Pharr, “Elastic and Plastic Characteristics of Sodium Metal,” ACS Applied Energy Materials 3, no. 2 (2020): 1759–1767. https://doi.org/10.1021/acsaem.9b02225

[10] C. Liu, X. Fu, S. Liao, G. Zou, and H. Yang, “Interface Engineering Enables High-Performance Sb Anode for Sodium Storage,” Nanomaterials 13, no. 2 (2023): 254. https://doi.org/10.3390/nano13020254

[11] J. Wang, C. Cen, X. Chen, et al., “Constructing a Na_3_Sb-Incorporated Solid Electrolyte Interphase by Rational Composite Electrode Design for Stable Sodium Metal Batteries,” Nano Letters (2026). https://doi.org/10.1021/acs.nanolett.6c01023
